# Mixed Anæsthesia by Ether, Morphine, and Atropine

**Published:** 1883-06

**Authors:** 


### Mixed Anæsthesia by Ether, Morphine, and Atropine.

Under the above title, M. P. Aubert, of Lyons (*Lyon Medicale*, 14 Janvier, 1883), discusses the question of the conjoint administration of ether or chloroform, morphine, and atropine. There are some very interesting subjects broached by Aubert, and the results of his experience are worthy of attentive consideration.

Those of our readers who have followed the history of anæsthesia, will recall the fact that Boston and Lyons, since the discovery of insensibility by inhalation of vapors, have remained loyal to ether as the best of the various agents hitherto brought forward for this purpose. Aubert, a representative of the Lyons sentiment, is not unmindful of the position of chloroform as an anæsthetic agent. His opinion is, therefore, the more valuable since he does not ignore the utility of other agents, although an advocate of ether. He also refers to the method of inducing the anæsthetic state, lately brought forward by M. Paul Bert, which consists in the administration, under pressure, of a mixture of oxygen and nitrous oxide; but this method, although strictly physiological, is objectionable because of the complexity of the apparatus required for its proper execution, and hence it is not feasible under the ordinary circumstances requiring the administration of an anæsthetic. To attain the maximum of safety with the anæsthetics now available is the problem. How best to secure the safe administration of ether or chloroform is, therefore, the point for consideration. M. Aubert maintains that the best results are had from the method of "mixed anæsthesia." This is the outcome of his experience, and of the collective observation of the Lyons surgeons. He calls attention to the fact that so long ago as 1878, Brinon, one of his pupils, presented a prize thesis on the anæsthesia obtained by the combined action of chloroform and morphine. During the following year, M. Hortholes demonstrated that the combination of ether and morphine was superior in respect to the promptness of the anæsthetic action, and the relief to the after-vomiting, to chloroform alone, or to chloroform and morphine. In 1882, M. Morat communicated to the profession the result of experiences he had acquired, in conjunction with M. Dastre, regarding the combined action of an anæsthetic with the subcutaneous injection of morphine and atropine. The theoretical view which led to this combination may be stated as follows:

Vulpian has shown that the excitability of the pneumogastric is increased by anæsthetic agents—whence the vomiting, and sometimes cardiac arrest. Now, morphine, whilst it increases the anæsthetic action, does not to any considerable extent lessen the effect on the pneumogastric nerve; but atropine, by removing the inhibition exerted by the vagus, removes the most important source of danger.

The injection of the solution of the combined morphine and atropine is practiced twenty or thirty minutes before the administration of the anæsthetic—usually ether. The ordinary dose of morphine is from one-twelfth to one-fourth of a grain, and of atropine from one one-hundred and eightieth to one-ninetieth of a grain. Complete insensibility is obtained in from three to seven minutes. The assistants, says Dr. Aubert, have been much surprised to note the difference which exists between the method of mixed anæsthesia--so calm and silent--and the inhalation of ether alone, with its period of excitement, and the after-vomiting and depression.

The great superiority of ether over chloroform, as respects safety, is certain; but the unpleasant effects of the former, and the prolonged stage of excitement produced by its inhalation, constituted almost insuperable objections to its use. M. Aubert now maintains that the method of mixed anæsthesia obviates these objections, so that ether may be given with the same ease and satisfaction as chloroform.

In view of the recent sad examples, illustrating the danger of chloroform anæsthesia, it is the more desirable to be put in possession of a method which combines the facility of chloroform inhalations with the superior safety of ether. In the method of "mixed anæsthesia," this desirable result seems attained.

